# Uneven Global Distribution of Theranostics

**DOI:** 10.1055/s-0046-1824537

**Published:** 2026-06-15

**Authors:** Kalevi Kairemo, Akram Al-Ibraheem

**Affiliations:** 1 Department of Theranostics, Docrates Cancer Center, Helsinki, Finland; 2 Department of Nuclear Medicine, King Hussein Cancer Center (KHCC), Amman, Jordan; 3 School of Medicine, University of Jordan, Amman, Jordan

Theranostics—especially nuclear medicine-based theranostics such as positron emission tomography (PET) imaging paired with targeted radionuclide therapy (e.g., ^177^ Lu-PSMA and ^177^ Lu-DOTATATE)—is currently distributed very unevenly across the world. The strongest clinical infrastructure and highest treatment availability are concentrated in high-income regions, including North America, Western Europe, and Australia. These countries generally have advanced PET/CT networks, cyclotrons, radiopharmacies, trained personnel, and reimbursement systems that support routine theranostic care. 1

In stark contrast, many low- and middle-income countries (LMICs) have limited or no access to theranostic services. Large parts of Africa, Latin America, and some regions of Asia lack sufficient PET scanners, radionuclide supply chains, radiochemistry facilities, and specialized workforce capacity. According to international surveys, low-income countries reported almost no use of modern prostate specific membrane antigen (PSMA)—or somatostatin receptor-targeted radionuclide therapies. 1 2

Globally, access is influenced by several practical factors: Availability of PET/CT and SPECT scanners, access to isotopes such as Lutetium-177 and Gallium-68, radiopharmaceutical manufacturing capacity, trained nuclear medicine staff, regulatory approval pathways, and reimbursement policies. 2 Beyond infrastructure limitations, the implementation of radiotheranostics is increasingly challenged by complex economic, regulatory, logistical, and geopolitical barriers. 3 Recent international analyses have highlighted substantial disparities in access to radionuclide production facilities, trained multidisciplinary workforce, reimbursement systems, quality management programs, and sustainable radiopharmaceutical supply chains, particularly across LMICs. 3 These inequalities create what may be described as a “theranostic affordability paradox,” whereby many countries possess significant clinical demand and expertise but remain constrained by the high costs of imported isotopes, fragmented regulatory pathways, and insufficient local production capacity. Addressing these barriers requires coordinated international strategies focused on education and training, regional isotope production networks, research collaboration, and implementation of robust quality and dosimetry frameworks. 3 4

International organizations such as the International Atomic Energy Agency (IAEA) are trying to reduce these disparities through training programs, infrastructure support, and global databases like IMAGINE, which track nuclear medicine capacity worldwide. However, the field is still heavily concentrated in wealthier health care systems, and equitable global access remains a major challenge. 2

High-income countries often have 10 to 40 PET scanners per million people, while many low-income countries have fewer than 1 scanner per million, and some have none at all. For example, the United States, Germany, and Australia have dense PET/CT networks, whereas several African countries operate only a single PET center nationally. 1 2

Europe currently performs some of the world's highest volumes of radioligand therapy. Germany alone has reported tens of thousands of PSMA PET scans annually and hundreds of certified theranostic treatment sites. In contrast, many countries in sub-Saharan Africa have no local Lutetium-177 therapy programs and depend on overseas referral or imported doses. The IAEA estimated that approximately 70% to 80% of global nuclear medicine procedures occur in North America, Europe, Japan, and Australia, while regions containing over half of the world's population account for only a small fraction of theranostic treatments.

Workforce differences are also substantial: High-income countries may have 10 to 20 nuclear medicine physicians per million inhabitants, whereas many LMICs have fewer than 1 per million. Similar shortages exist for radiopharmacists, medical physicists, and technologists.

Access to approved therapies differs sharply: ^177^ Lu-DOTATATE and ^177^ Lu-PSMA are reimbursed and routinely used in countries such as Germany, the United States, and Australia. In many African and lower-income Asian countries, patients may wait months for imported isotopes or travel internationally for treatment.

The global landscape of theranostic readiness and infrastructure can be broadly categorized as follows:

**Table:** 

Region	PET access	Theranostic treatment availability
North America/Western Europe	High (10–40+ PET scanners per million)	Clinical routine
Japan/South Korea/Australia	High	Clinical routine
Latin America	Moderate, available in major cities	Limited but increasing
Middle East	Variable	Growing rapidly in some countries
South and Southeast Asia	Uneven	Available in some tertiary centers
Sub-Saharan Africa	Very low	Sparse or absent in many countries

The field is expanding quickly, but current practice is still strongly correlated with national health care spending, isotope production infrastructure, and nuclear medicine training capacity.

The *World Journal of Nuclear Medicine* addresses this uneven global distribution of theranostics in an IAEA article. 2 To illustrate this phenomenon in practice, in this issue, there is an example of an Australian phase I clinical trial 5 and another about the use of nuclear medicine in Nepal. 6 The published case reports in this issue demonstrate wide local spread, and clinical examples with literature are presented from four continents. All these contributions are important for our community.

Radiotheranostics is progressively evolving from a specialized nuclear medicine application into a central pillar of precision oncology and personalized medicine. Although the field is expanding rapidly worldwide, current practice remains strongly correlated with national health care expenditure, isotope production infrastructure, regulatory maturity, and workforce capacity. These observations are aligned with growing international recommendations emphasizing the importance of equitable global expansion of radiotheranostics capacity, particularly in underserved and resource-limited regions. Sustainable global expansion will require stronger international collaboration, investment in local radiopharmaceutical production, harmonized training initiatives, robust quality management systems, and equitable implementation strategies. Importantly, broader inclusion of LMICs in international prospective phase III clinical trials is important to ensure equitable evidence generation, improve global applicability of clinical data, strengthen regional expertise, and accelerate sustainable implementation across diverse health care settings. Ensuring broader access to radiotheranostics should become a global health care priority rather than a privilege restricted to highly resourced health care systems.
